# Central venous pressure monitoring and mortality: What was neglected?

**DOI:** 10.1186/s13054-020-03350-2

**Published:** 2020-10-23

**Authors:** Shaowei Gao, Zhanxin Du, Lu Yang, Zhongxing Wang

**Affiliations:** Department of Anesthesia, The First Affiliated Hospital of Sun Yat-Sen University, 58 Zhongshan 2nd Road, Guangzhou, 510080 China

**Dear editor**,

We are interested in the recent published article about the association between central venous pressure (CVP) monitoring and mortality for ICU patients with sepsis [1]. The study provides new insights into this traditional monitoring. However, an important factor might make the study more convincing if it had been taken into account.

The clinical experience tells us that ICU admissions after surgeries (surgical patients in the ICU) are more likely to have central venous catheters than those from the medical system (medical patients in the ICU). Besides, as has been proven by many studies, medical patients have worse prognosis than surgical patients in the ICU [2–4]. Chen and his colleagues collected tens of important covariates to adjust the results, but the admission resource (from surgical or medical systems, which can be identified with the official codes [5]) was neglected [1]. To validate our supposition, we conducted an analysis in the same database. According to the inclusion and exclusion criteria of Chen’s study, we extracted a very similar (10,131) but not identical (10,275) cohort (because we didn’t get the authors’ original codes). The CVP monitoring group has 4,505 patients (vs. 4516 in Chen’s study), while the non-CVP monitoring group has 5626 ones (vs. 5759 in Chen’s study). As shown in the mosaic plot (Fig. 1a), CVP monitoring is positively associated with ICU admissions after surgery (1574/4505 [35%] for CVP group vs 835/5626 [15%] for non-CVP group, Phi coefficient = 0.235, p < 0.001). And the 28-day mortality is significantly lower among surgical patients (12% for surgical patients vs 21% for medical patients, relative risk [95% confidence interval]: 0.60 [0.53–0.67], p < 0.001). Briefly, the CVP monitoring group has a larger proportion (more than twice the non-CVP monitoring group) of surgical patients, which has a lower 28-day mortality rate (nearly a half) than medical patients. Not considering the admission resources may bring bias to Chen’s study.

**Fig. 1:**
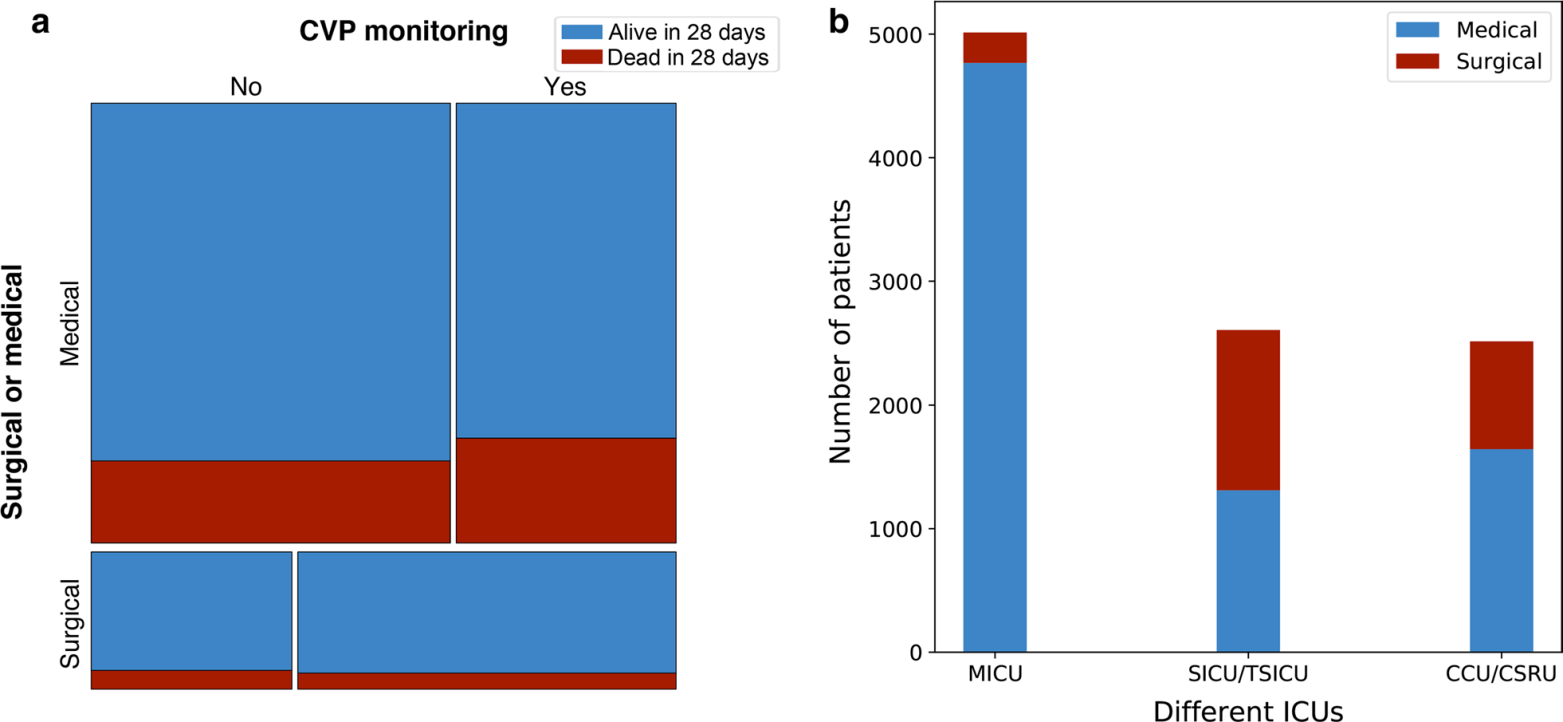
**a** The mosaic plot for the association of CVP monitoring and Admission resources (from surgical or medical systems). The area of each block refers to the number of patients for each category. These categories are marked horizontally by CVP monitoring and vertically by Admission resources. The red portion of each block refers to 28-day mortality. **b** The bar chart of numbers of patients in different ICUs. The numbers of all, surgical and medical patients can be identified for each kind of ICUs. *CVP* central venous pressure, *ICU* intensive care unit, *MICU* medical intensive care unit, *SICU* surgical intensive care unit, *TSICU* trauma surgical intensive care unit, *CCU* coronary care unit, *CSRU* cardiac surgery unit

Service units of patients in the MIMIC database (MICU, SICU/TSICU and CCU/CSRU in Chen’s study) were collected as a covariate. Except for MICU, the other two units were balanced in the numbers of surgical and medical patients (Fig. 1b). As a result, the proportions of surgical and medical patients could not be adjusted between CVP monitoring and non-CVP monitoring groups, which means that the effect of admission resources could not be replaced by service units. We would be very interested in the results if the effect of admission resources were considered.

## Data Availability

The datasets extracted and analyzed during the current study are accessed in this website (https://mimic.physionet.org/gettingstarted/access/).
